# Application of tip-flexible vacuum-assisted ureteral access sheath in flexible ureteroscopic laser lithotripsy for renal stones in a child after pyeloplasty: a case report

**DOI:** 10.3389/fped.2025.1525840

**Published:** 2025-04-11

**Authors:** Qiang Cheng, Jianwei Cao, Lin Zhang, Qiaolin Chen, Houwei Lin

**Affiliations:** ^1^ Department of Pediatric Urology, Xinhua Hospital Affiliated to Shanghai Jiaotong University School of Medicine, Shanghai, China; ^2^ Department of Urology, Xinhua Hospital Affiliated to Shanghai Jiaotong University School of Medicine, Shanghai, China; ^3^ Department of Pediatric Urology, Zhejiang Chinese Medical University, Hangzhou, Zhejiang, China

**Keywords:** tip-flexible vacuum-assisted ureteral access sheath, retrograde intrarenal stone surgery, kidney stones, pediatric lithotripsy, ureteropelvic junction obstruction

## Abstract

**Objectives:**

To explore the application of tip-flexible vacuum-assisted ureteral access sheath-assisted retrograde intrarenal stone surgery/flexible ureteroscopic lithotripsy in children.

**Patients and methods:**

A retrospective analysis was conducted on the clinical data of a pediatric patient who developed kidney stones following pyeloplasty for congenital ureteropelvic junction obstruction. The child underwent tip-flexible vacuum-assisted ureteral access sheath-assisted retrograde intrarenal stone surgery under general anesthesia. Inpatient records and postoperative follow-up results were collected.

**Results:**

The kidney stones were utterly removed, and there were no complications like infection, bleeding, or secondary obstruction. Moreover, there was no significant damage to the anastomotic site after pyeloplasty.

**Conclusion:**

The use of tip-flexible vacuum-assisted ureteral access sheath-assisted flexible ureteroscopic lithotripsy is safe and effective for pediatric lithotripsy.

## Introduction

Congenital Ureteropelvic Junction Obstruction (UPJO) is a common ureteral anomaly, occurring in approximately 1 in 750–1,500 newborns (1). UPJO impedes normal urinary excretion, leading to upper urinary tract dilatation and renal functional impairment, which predispose to calculus formation (2). Pyeloplasty is the standard treatment for UPJO, with a success rate of nearly 90% (3). Nonetheless, even after pyeloplasty, kidney stones may still develop, associated with renal dysfunction, metabolic disorders, relative stenosis at the junction, and other factors (2, 4).

Compared with percutaneous nephrolithotomy (PCNL) or extracorporeal shock wave lithotripsy (ESWL), retrograde intrarenal surgery (RIRS) or flexible ureteroscopic lithotripsy (FURS) has emerged as the preferred treatment for upper urinary tract stones smaller than 20 mm. This approach uses a flexible ureteroscope, laser lithotripsy equipment, and a ureteral access sheath (UAS). RIRS is favored due to its high stone-free rate (SFR), low complication rate, and minimally invasive nature. Additionally, it avoids the repeated disconnection of the pelviureteric junction (5–7). A novel type of UAS, the tip-flexible vacuum-assisted ureteral access sheath (FV-UAS), features excellent flexibility and deformability at its tip, allowing it to passively bend in sync with the FURS (8). Wei Zhu et al. (8) demonstrated that FV-UAS provides significant safety and efficacy in adults. This article mainly demonstrates RIRS combined with FV-UAS in the treatment of urinary lithiasis after pyeloplasty for UPJO in a child.

## Case report

### Medical history

**Figure 1:**
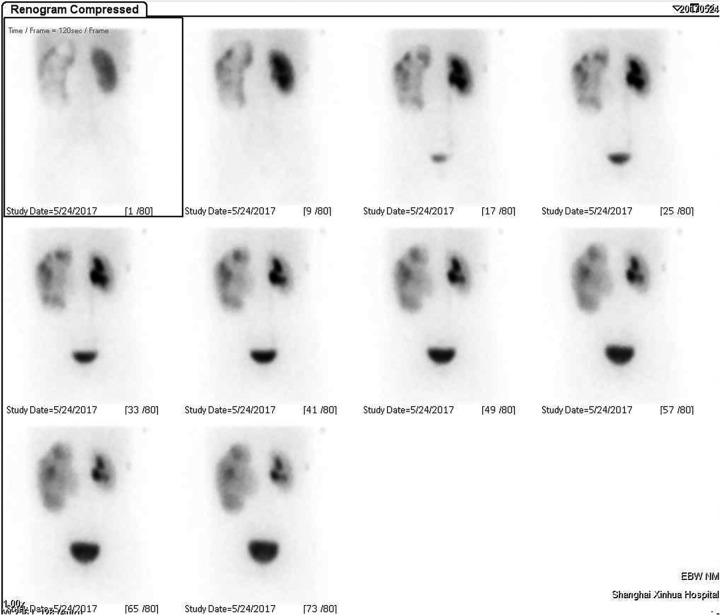
The scintigraphy before pyeloplasty.

**Figure 2:**
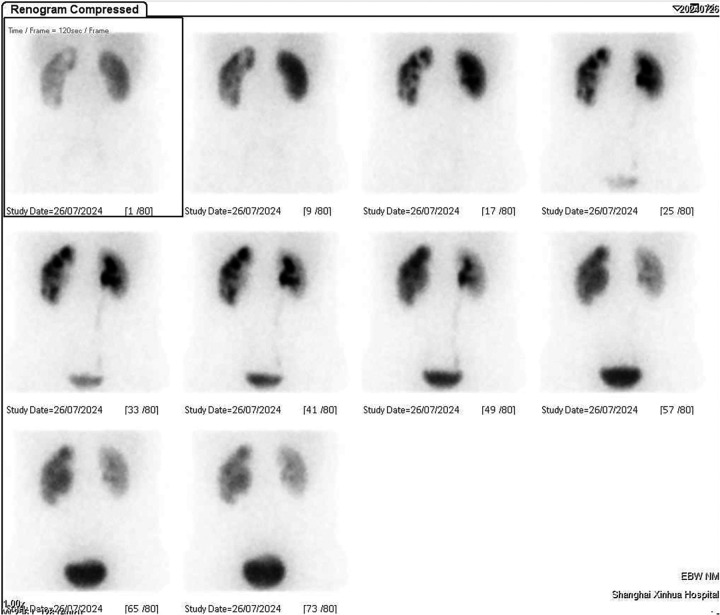
The scintigraphy during follow-up after pyeloplasty.

A 13-year-old boy was admitted to Xinhua Hospital, affiliated with Shanghai Jiaotong University School of Medicine, with a history of left upper urinary tract dilation for seven years and newly diagnosed left kidney stones detected two weeks prior. The child was diagnosed with dilatation of the left upper urinary tract at 28 weeks of gestation. Within one year after birth, the left renal pelvis separation was 10–20 mm according to ultrasonography (US). During the follow-up in our hospital in May 2017 (when the child was 6 years old), the US indicated the aggravation of dilatation of the left upper urinary tract: the anteroposterior diameter of the left renal pelvis measured 54 mm, and all renal calyces were dilated, with the upper calyx separation of 49 mm and cortical thickness of 1.8 mm. Renal dynamic imaging showed obvious dilatation and thinning of the left renal cortex, with renal function of 20.82%, and mechanical obstruction of the left upper urinary tract (Figure 1). In June 2017, the child underwent left pyeloplasty in our hospital due to suspected left ureteropelvic junction stenosis. Postoperative follow-up of the child demonstrated a gradual reduction in the anteroposterior diameter of the left renal pelvis to approximately 25 mm, with subsequent stabilization maintained throughout subsequent monitoring. Renal dynamic imaging showed that the left renal function increased to 39.25%, and the excretion function of the upper urinary tract improved (Figure 2). On July 24, 2024, a routine ultrasound examination of the child revealed that the anteroposterior diameter of the left renal pelvis measured 26 mm, and an 11 mm stone was detected in the left lower calyx. The child exhibited no symptoms such as fever, hematuria, proteinuria, pyuria, lumbar pain, abdominal pain, frequent urination, or dysuria.

### Surgical strategy and techniques

**Figure 3:**
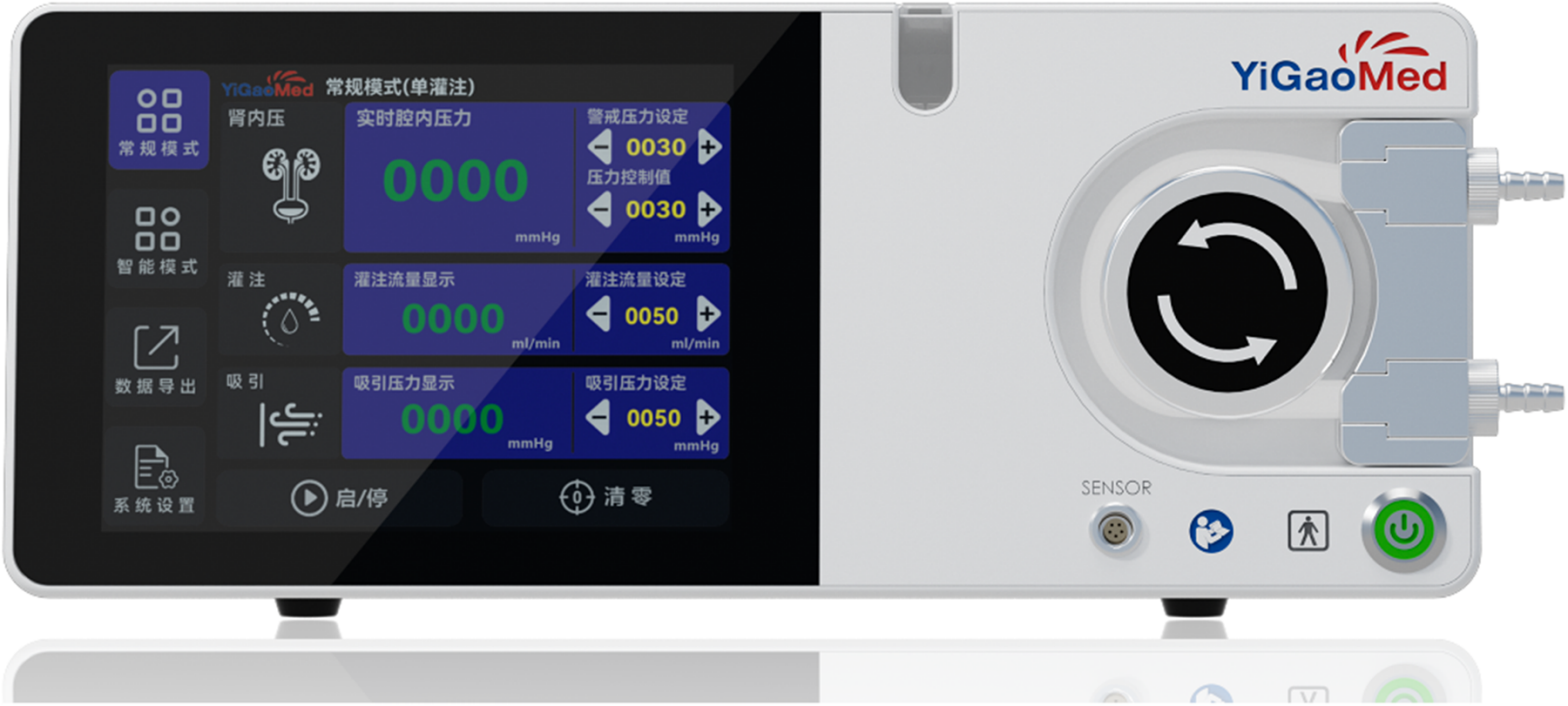
Controlled pressure machine.

The surgical measure was the FV-UAS-assisted retrograde intrarenal stone surgery and the anesthesia method was general anesthesia. In the lithotomy position, an F6/7.5 Wolf ureteroscope was inserted. The left ureteral orifice was located, and a guidewire (COOK®, 0.035 inches, America) was inserted into the ureter. Advancing the ureteroscope upwards along the guide wire, the distorted upper section of the ureter and surgical scars at the pyeloureteral anastomosis site were observed, with a slightly narrower lumen. After the guidewire was retained, a 50 cm F11/13 FV-UAS (YiGaoMED®, China) was inserted along the guidewire and an F8.4 disposable FURS (INNOVATE®, China) entered into renal pelvis calyces along the sheath. A perfusion pump (Shiyin Medical®, China) was used for perfusion with a 300 mmHg perfusion pressure and a 0.5 L/min flow. The −30 KPa negative pressure was connected to the sheath negative pressure port for suction (YiGaoMED®, China) (Figure 3). During the operation, multiple stones were found in the inferior renal calyx, yellowish-white in color, with the largest one measuring approximately 0.8 cm in length. The stones were fragmented by means of a holmium laser of 1.0J, 20 Hz (Lumenis®, America), and the stone fragments were aspirated by the FV-UAS. The ureteroscope was withdrawn, and a double-J stent was inserted into the left ureter over the guidewire, which was to be indwelt for 3 weeks. Finally, an F12 double-lumen Foley catheter was inserted, and the balloon was filled with 20 ml of water.

## Results

**Figure 4:**
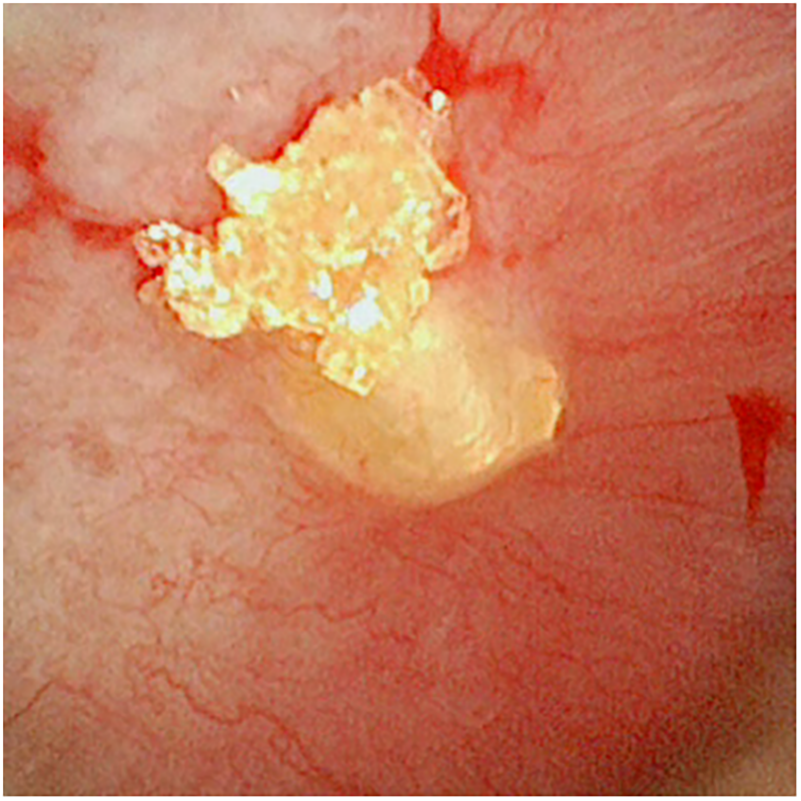
Submucosal stones near the middle of the left inferior renal calyx.

Intraoperatively, not only were the stones in the inferior calyx of the left kidney located, but also submucosal stones aggregation near the middle calyx was identified (Figure 4), showing a history of stones formation and a potential risk of metabolic abnormalities in the child. Laser lithotripsy was utilized to fragment the stones, and the majority of the stone fragments were removed (Supplementary Video S1). Postoperative stone analysis revealed a mixed composition (60% protein, 40% anhydrous uric acid).

Upon postoperative examination with the FURS, the anastomosis was well and there was no obvious damage to the ureter. The US revealed no significant abnormal echoes in the kidneys, ureters, or bladder, no obvious dilation of the bilateral ureters and no progression of dilatation of the left upper urinary tract two days after surgery. The child was prophylactically administered Cefuroxime Sodium 1.5 g bid within 48 h postoperatively. The patient remained asymptomatic without fever, hematuria, or worsening dilation and was discharged in stable condition.

## Discussion

For patients with UPJO accompanied by stones, open or minimally invasive pyeloplasty is the preferred treatment, which can achieve a high SFR while relieving obstruction (9–11). However, given that the child has undergone pyeloplasty and the anastomosis at the pyeloureteric junction is well, repeated damage to the pyeloureteric junction may lead to secondary postoperative stenosis. Moreover, based on the European Association of Urology guidelines (12), for renal stones less than 20 mm in the lower calyx or non-lower calyx, RIRS is considered the first-line treatment option. Moreover, potential indications of RIRS include anatomic abnormalities of the urinary system, multiple kidney stones, bleeding disorders, ancillary procedures after PCNL, obesity etc. There is no specific contraindication for RIRS, except an untreated urinary tract infection and other anesthesia contraindications (13). Therefore, RIRS is selected. FURS and FV-UAS enter the kidney through the urethra, bladder, and ureter, which is a non-invasive operation and avoids repeated disconnection of the pyeloureteric junction. In addition, compared with other lithotripsy surgeries, De Shuba et al. (14) indicated that although the SFR of RIRS is lower than that of PCNL, the probability of complications such as bleeding, infection, and fever and hospital stays after PCNL are significantly higher than those of RIRS. For stones less than 2 cm, Arif Demirbas et al. (15) thought the SFR of RIRS is higher than that of minimally PCNL. Moreover, ESWL may lead to stone excretion difficulty, causing stone accumulation at the stenosis and secondary obstruction.

During RIRS, the UAS directly establishes renal access and is connected to negative pressure suction. After laser lithotripsy, stone fragments can be expelled through the gap between the UAS and the FURS along with the irrigation fluid, or with the irrigation fluid after withdrawing the FURS. However, the traditional rigid UAS has poor flexibility and bending capabilities, and is generally placed at the pyeloureteric junction, resulting in poor regulation of intrarenal pressure. During RIRS, increased intrarenal pressure may cause urine reflux and absorption, leading to severe infections. The FV-UAS is a novel type of UAS featuring excellent flexibility and deformability at its tip. Similarly, it can be connected to a negative pressure suction device. The FV-UAS can pass the pyeloureteric junction alongside the FURS, and under its guidance, the FV-UAS can approach the stone as closely as possible, accurately sucking up stone fragments while maintaining a low intrarenal pressure through negative pressure suction. This reduces the risk of urine reflux (8, 16).

Many clinical studies have proved the effectiveness of RIRS combined with FV-UAS. Yujun Chen et al. (17) found that FV-UAS can achieve complete stone clearance without the need to use stone baskets to reduce costs, and FV-UAS can maintain a low intrarenal pressure under the condition of high-flow rate irrigation fluid, which can reduce the occurrence of complications while ensuring a good surgical field of vision and high clearance rate. As for traditional UAS, clinical researches (18) indicate that the effectiveness of RIRS with traditional rigid UAS is not ideal, with SFR ranging from 50% to 90% in adult patients during the initial surgery, and 50% to 92% in pediatric patients. Ito et al. (19) thought that dilation of the renal pelvis and renal calyces could increase the difficulty of lithotripsy and stone basket removal, and the possibility of residual stones after surgery. Baiyang Song et al. (20) also demonstrated through *in vitro* experiments that severe upper urinary tract dilatation significantly impairs stone excretion. Moreover, when dealing with the inferior caliceal stones, the bending degree of FURS is so limited within the traditional rigid UAS, resulting in a low SFR of RIRS that Unsal et al. (21) once believed that PCNL should be the first choice for the treatment of lower caliceal stones larger than 10 mm. However, Deheng Cui et al. (22) proved that FV-UAS has significant advantages in SFR by virtue of its bendable head, and Gaoyuanzhi Yue et al. (23) used FV-UAS to treat an 11 mm stone in the left inferior renal calyx of a female patient, achieving the 100% SFR without the assistance of a stone basket and without any complications occurring. In addition, Wei Zhu et al. (8) also pointed out that the process of placing FV-UAS is smoother with less resistance, causing less damage to the ureter compared to traditional UAS. Hui Liang et al. (24) conducted a retrospective analysis of 244 stone patients treated with RIRS combined with FV-UAS, and only 2 cases developed fever symptoms. Therefore, RIRS combined with FV-UAS can achieve a higher SFR and lower complication rate, but it should be noted that FV-UAS may still cause fluctuations and sudden increases in intrarenal pressure, leading to serious complications such as renal abscess (25), so regulating intrarenal pressure is an important factor in reducing postoperative complications of RIRS.

In this study, the child exhibited significant deformation and tortuosity of the renal pelvis and calyces, making it more challenging to explore as many angles as possible. The RIRS combined with FV-UAS ensures a good surgical field of vision with lower intrarenal pressure. This approach allows for the detection of hidden stones in the long and narrow, curved calyces. Moreover, faced with deformed and narrowed calyces, if a traditional rigid UAS were used, relying solely on irrigation fluid to flush the stones into the renal pelvis and ureter would increase the likelihood of stone residue due to generating vortexes. In contrast, the targeted suction of FV-UAS avoids this risk, achieving a higher SFR. Furthermore, the smoother and softer FV-UAS can easily pass through narrow segments of the ureter and operate in the broader renal pelvis, reducing the risk of damage to the ureteral wall and avoiding secondary obstruction at the anastomosis site post-lithotripsy.

In summary, the FV-UAS is a crucial auxiliary tool and RIRS combined with FV-UAS can ensure a high SFR and reduce complications in a non-invasive manner, and can also be used in cases of ureteral stenosis. However, maintaining low intrarenal pressure is essential. Therefore, for children with secondary urinary stones after pyeloplasty for congenital UPJO, RIRS combined with FV-UAS is an effective, safe, and non-invasive surgical approach.

## Data Availability

The original contributions presented in the study are included in the article/Supplementary Material, further inquiries can be directed to the corresponding author.
